# Fermentation of oxidized hexose derivatives by *Clostridium acetobutylicum*

**DOI:** 10.1186/s12934-014-0139-7

**Published:** 2014-09-18

**Authors:** Matthew D Servinsky, Sanchao Liu, Elliot S Gerlach, Katherine L Germane, Christian J Sund

**Affiliations:** US Army Research Laboratory, Sensors and Electron Devices Directorate, 2800 Powder Mill Road, Adelphi, MD 20783 USA; Federal Staffing Resources, 2200 Somerville Rd, Annapolis, MD 21401 USA; Oak Ridge Associated Universities, 4692 Millennium Drive, Suite 101, Belcamp, Maryland 21017 USA

**Keywords:** *Clostridium acetobutylicum*, Fermentation, Gluconate, Galacturonate, Acetate, Pectin, Hydrogen, Carbon dioxide

## Abstract

**Background:**

*Clostridium acetobutylicum* fermentations are promising for production of commodity chemicals from heterogeneous biomass due to the wide range of substrates the organism can metabolize. Much work has been done to elucidate the pathways for utilization of aldoses, but little is known about metabolism of more oxidized substrates. Two oxidized hexose derivatives, gluconate and galacturonate, are present in low cost feedstocks, and their metabolism will contribute to overall metabolic output of these substrates.

**Results:**

A complete metabolic network for glucose, gluconate, and galacturonate utilization was generated using online databases, previous studies, genomic context, and experimental data. Gluconate appears to be metabolized via the Entner-Doudoroff pathway, and is likely dehydrated to 2-keto-3-deoxy-gluconate before phosphorylation to 2-keto-3-deoxy-6-P-gluconate. Galacturonate appears to be processed via the Ashwell pathway, converging on a common metabolite for gluconate and galacturonate metabolism, 2-keto-3-deoxygluconate. As expected, increasingly oxidized substrates resulted in increasingly oxidized products with galacturonate fermentations being nearly homoacetic. Calculations of expected ATP and reducing equivalent yields and experimental data suggested galacturonate fermentations were reductant limited. Galacturonate fermentation was incomplete, which was not due solely to product inhibition or the inability to utilize low concentrations of galacturonate. Removal of H_2_ and CO_2_ by agitation resulted in faster growth, higher cell densities, formation of relatively more oxidized products, and higher product yields for cultures grown on glucose or gluconate. In contrast, cells grown on galacturonate showed reduced growth rates upon agitation, which was likely due to loss in reductant in the form of H_2_. The growth advantage seen on agitated glucose or gluconate cultures could not be solely attributed to improved ATP economics, thereby indicating other factors are also important.

**Conclusions:**

The metabolic network presented in this work should facilitate similar reconstructions in other organisms, and provides a further understanding of the pathways involved in metabolism of oxidized feedstocks and carbohydrate mixtures. The nearly homoacetic fermentation during growth on galacturonate indicates further optimization of this and related organisms could provide a route to an effective biologically derived acetic acid production platform. Furthermore, the pathways could be targeted to decrease production of undesirable products during fermentations of heterogeneous biomass.

## Background

Slop food waste is a substantial portion of total waste from civilian and military culinary operations [1,2]. The high water content of slop food waste makes it a poor substrate for combustion based waste to energy conversions. Therefore, to achieve maximal energy efficiency and on-site waste mitigation, alternate technologies need to be explored. One promising possibility is fermentation of slop food waste, which is rich in carbohydrates and other nutrients, to fuels such as butanol or ethanol. The anaerobic bacterium *Clostridium acetobutylicum* is an excellent candidate to perform this task due to its abilities to use a wide variety of carbohydrates and to produce fuels in the form of hydrogen gas, ethanol, and butanol [3-5]. *C. acetobutylicum* has been used at the industrial scale for production of the solvents acetone, butanol, and ethanol from plant based starches [4,6,7]. To optimize fermentation of slop food wastes, which are heterogeneous, it is necessary to establish a thorough understanding of how carbohydrates found in food are metabolized, and their contribution to metabolic output.

Two major factors controlling metabolic output of fermentations are the redox state of the feedstock and the pathway used for metabolism [8-10]. Slop food waste contains a vast array of carbohydrates and their derivatives, some of which are more oxidized than the hexoses commonly used in the study of *C. acetobutylicum*’s metabolism [11-13]. Two such carbohydrate derivatives are gluconate and galacturonate [11,12]. Gluconate is more oxidized than glucose by 2 electrons, and is found in fruit, honey, rice, meat, and other foods [11]. Galacturonate is more oxidized than glucose by 4 electrons, and is the primary constituent of pectin [12]. While all plant cell walls contain pectin, many foods such as fruit are enriched for this complex carbohydrate [12]. Since both of these carbohydrate derivatives are enriched in food, their contribution to metabolic output is especially important for food waste fermentation.

Numerous clostridial species are capable of fermenting galacturonate and gluconate, but little is known about the pathways and the associated genes contributing to this process in solventogenic clostridia [14-18]. An early study comparing *C. acetobutylicum* fermentations on substrates with varying degrees of oxidation showed fermentation of gluconate resulted in higher acetate:butyrate ratios when compared to glucose fermentations [10]. Additionally, acetone was the predominant solvent produced during solventogenesis for gluconate fermentations, while butanol was the main solvent produced in glucose fermentations [10]. Other studies showed *C. aceticum, C. formicoaceticum, C. butyricum, C. pasteurianum, C. roseum, and C. butylicum* fermented gluconate via the Entner-Doudoroff (ED) pathway, but no evidence has verified that *C. acetobutylicum* uses the ED for gluconate metabolism [14,18]. To the best of our knowledge the only *Clostridium* species where a complete pathway for galacturonate utilization has been experimentally confirmed is *C. thermosaccharolyticum,* which uses the Ashwell pathway (also called the Modified Entner-Doudoroff pathway, 2-keto-3-deoxy-6-phospho-D-gluconate (KDPG) pathway, or isomerase pathway) to metabolize galacturonate in a similar manner to *Escherichia coli* and *Erwinia chrysanthemi* [19-22]. While it is common knowledge that fermentation of increasingly oxidized substrates results in increasingly oxidized products, to our knowledge there is only one report describing the metabolic output of a solventogenic bacterium during growth on galacturonate, and it revealed *C. butryricum* produced predominately acetate from polygalacturonate [23].

Bioinformatically driven metabolic network reconstructions for *C. acetobutylicum* by the genome annotation, BioCyc, and KEGG identified complete pathways for galacturonate metabolism via the Ashwell pathway [19,21]. However, these reconstructions either misidentified or did not identify genes responsible for gluconate metabolism [24-26]. This study provides insight into gluconate and galacturonate metabolism by using manual curation to reconstruct *C. acetobutylicum’s* gluconate utilization pathway, yields the first experimental evidence for gluconate utilization via the ED pathway, and examines metabolic output of fermentations of oxidized carbohydrate derivatives. The information provided by this study is useful for designing strategies to increase production of desired products from heterogeneous feedstocks such as food waste.

## Results and discussion

### Reconstruction of metabolic network

**Figure 1:**
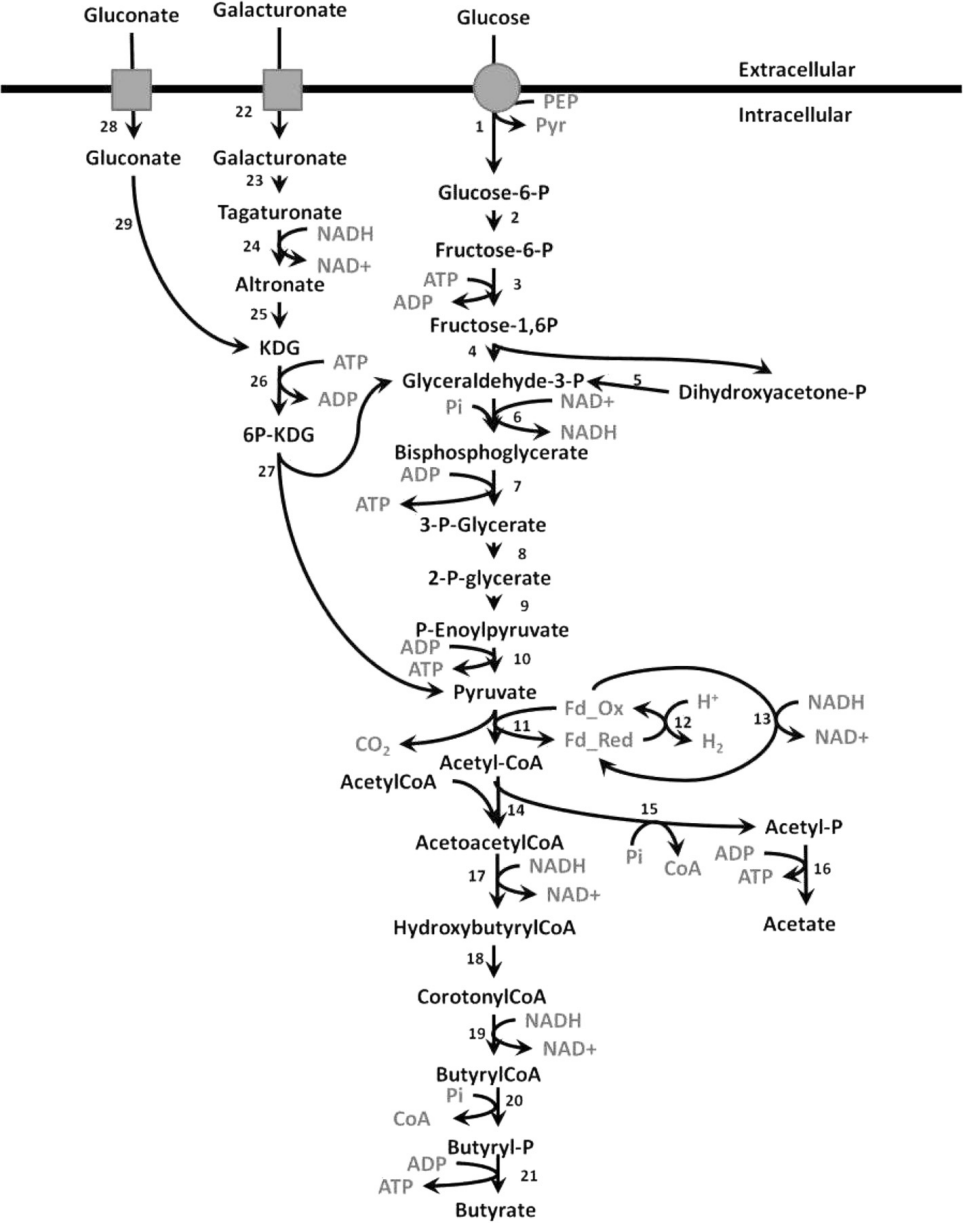
**Metabolic network of carbon source metabolism by** ***Clostridium acetobutylicum*** **reconstructed from the genome annotation, KEGG, and BioCyc.** Numbers adjacent to arrows represent the following enzyme activities and their corresponding genes. **1)** glucose PTS, CA_C0570; **2)** phosphoglucose isomerase, CA_C2680; **3)** phosphofructokinase, CA_C0517; **4)** fructose-bis-P aldolase, CA_C0827, CA_P0064; **5)** triosephosphate isomerase, CA_C0711; **6)** glyceradehyde-3-P dehydrogenase, CA_C0709; **7)** phosphoglycerate kinase, CA_C0710; **8)** phosphoglycerate mutase, CA_C0712, CA_C2741, CA_C3021; **9)** enolase, CA_C0713; **10)** pyruvate kinase, CA_C0518, CA_C1036; **11)** pyruvate ferredoxin oxidoreductase, CA_C2229, CA_C2499; **12)** hydrogenase, CA_C0028; **13)** NAD^+^/ferredoxin oxidoreductase, CA_C?; **14)** thiolase, CA_C2873, CA_P0078; **15)** phosphotransacetylase, CA_C1742; **16)** acetate kinase, CA_C1743; **17)** hydroxybutyryl-CoA dehydrogenase, CA_C2009, CA_C2708; **18)** crotonase, CA_C2012, CA_C2016, CA_C2712; **19)** butyryl-CoA dehydrogenase, CA_C2711; **20)** phosphotransbutyrylase, CA_C3076; **21)** butyrate kinase, CA_C1660, CA_C3075; **22)** galacturonate symporter, CA_C0694; **23)** galacturonate isomerase, CA_C0692 24) altronate oxidoreductase, CA_C0695; **25)** altronate dehydratase, CA_C0696; **26)** 2-keto-3-deoxygluconokinase, CA_C0395; **27)** 2-keto-3-deoxygluconate 6-phosphate aldolase, CA_C0394, CA_C2973; **28)** gluconate symporter, CA_C3605; **29)** gluconate dehydratase, CA_C3604.

The metabolic network for utilization of glucose, gluconate, and galacturonate shown in Figure 1 was reconstructed using manual curation, the genome annotation, MetaCyc, and KEGG [24-26]. Glucose and galacturonate utilization pathways were easily reconstructed and there was satisfactory agreement between databases. Conversely, for gluconate there were metabolic gaps present in both KEGG and the genome annotation, with the complete pathway in MetaCyc appearing to be misannotated. Both databases and the genome annotation predicted the presence of two gluconate/proton symporters encoded by the genes CA_C3605 and CA_C2835. In a previous study, it was shown that galactose induced CA_C2835 and genes for the Leloir pathway, thereby suggesting the symporter encoded by CA_C2835 is involved in uptake of unphosphorylated galactose for metabolism via the Leloir pathway [3]. Therefore, the most likely candidate for uptake of gluconate is the CA_C3605 gene product.

The pathway for gluconate metabolism in MetaCyc proceeded via the ED pathway, where phosphorylation of gluconate to 6-phosphogluconate by gluconokinase was followed by dehydration to KDPG by 6-phosphogluconate dehydratase. KDPG is a common intermediate for gluconate and galacturonate metabolism via the ED pathway. It is converted to pyruvate and glyceraldehyde-3-P by KDPG aldolase, which appears to be encoded by CA_C0394 or CA_C2973. MetaCyc predicted that the gene CA_C2612 encodes gluconokinase but this gene has been shown to be induced by pentoses and encodes a xylulose kinase [3,27]. Additionally, a recent phylogenetic analysis of FGGY carbohydrate kinases (family of evolutionarily related carbohydrate kinases with diverse function) failed to identify a gluconokinase in *C. acetobutylicum* so it is unlikely that gluconokinase is present in the organism [28]. MetaCyc also predicted that CA_C3170 encodes 6-phosphogluconate dehydratase, however the genomic context of the gene is inconsistent with that function. A BlastP analysis of the CA_C3170 translated amino acid sequence indicated it is a dihydroxyacid dehydratase, but the gene appears to be in an operon with other genes for synthesis of valine, leucine, and isoleucine, which suggests its primary role is synthesis of amino acids [29]. The above information reconfirms that the automated methods used by MetaCyc and KEGG are often insufficient for predicting the gene product function, as is the case for the gluconate utilization pathway of *C. acetobutylicum*.

Two possible fates for intracellular gluconate, besides phosphorylation, are reduction to glucose or dehydration to 2-keto-3-deoxy-gluconate (KDG) [11]. The resulting glucose would most likely be metabolized via glycolysis, while KDG would be metabolized via the ED pathway. To determine which pathway was utilized for gluconate metabolism, cells were grown on D-[1-^13^C] gluconate, and NMR was used to measure ^13^C incorporation into fermentation products in a similar manner to a previous study of glucose metabolism in *Pyrococcus furiosus* [30]. If the cells converted gluconate to glucose, then ^13^C would have been present in the methyl group of pyruvate and acetyl-CoA after conversion of the carboxylic acid carbon of pyruvate to CO_2_ by the pyruvate:ferredoxin oxidoreductase (PFOR). Conversely, if the cells utilized the ED pathway for gluconate metabolism, then ^13^C would have been present in the carboxylic acid group of pyruvate, where it would have been converted to CO_2_. As a result ^13^C would not be found in the fermentation products. Analysis of media containing D-[1-^13^C] gluconate before and after fermentation by *C. acetobutylicum* indicated ^13^C was not incorporated into the fermentation products despite the detection of D-[1-^13^C] gluconate in the media prior to fermentation (data not shown). The results indicate gluconate is metabolized via the ED pathway, which is consistent with previous studies of gluconate metabolism in other *Clostridium* species [14,18]. The cells, therefore, most likely convert gluconate to KDG, requiring a gluconate dehydratase, which has not been identified in *C. acetobutylicum.* Upstream of the putative gluconate symporter gene is a gene, CA_C3604, annotated as encoding a putative IlvD type dihydroxyacid dehydratase. This class of enzymes includes gluconate dehydratase; the only other enzyme in this class for *C. acetobutylicum* is the xylulose kinase (CA_C2612), so we predict CA_C3604 encodes *C. acetobutylicum’s* gluconate dehydrogenase.

### Metabolic output

Table 1 shows the predicted yield of ATP, NADH, and reduced ferredoxin for metabolism of glucose, gluconate, and galacturonate to acetyl-CoA. It is clear from this that vast differences are present in the amount of reducing equivalents formed from the three substrates. Galacturonate fermentations may be starved for NADH and NADPH required for biosynthetic reactions and reduction of tagaturonate to altronate by altronate oxidoreductase. To determine how the formation of reducing equivalents affects metabolic output, cells were grown on glucose, gluconate, or galacturonate, and production of key metabolites was measured, as presented in Table 1. As expected, increasingly oxidized carbohydrates resulted in the formation of increasingly oxidized products as evidenced by comparing the acetate:butyrate ratios of the fermentations. Measured H_2_:CO_2_ ratios for glucose and gluconate were similar to those seen in previous reports [10,31]. The two main sources of reductant in *C. acetobutylicum* are NADH from lower glycolysis and reduced ferredoxin from PFOR. Electron carriers are reoxidized by the hydrogenase, which couples ferredoxin oxidation with proton reduction, and by reductive conversion of acetyl-CoA to butyrate. Cells gain one ATP per acetyl-CoA by conversion to acetate, as opposed to 0.5 ATP per acetyl-CoA generated during reduction to butyrate. It is therefore more favorable from the standpoint of ATP yield to use the hydrogenase to reoxidize electron carriers. Electrons can be shuffled between NADH and ferredoxin by the NADH-ferredoxin oxidoreductase, thus it is possible for reducing equivalents formed in lower glycolysis to be oxidized indirectly via the hydrogenase [31,32]. An excess of reductant was present in the glucose and gluconate fermentations and the hydrogenase was saturated, as indicated by H_2_:CO_2_ ratios greater than one and the substantial production of butyrate. In cases where H_2_:CO_2_ ratios were greater than one there likely was flow of electrons from NADH to ferredoxin via the NADH-ferredoxin oxidoreductase as reduced ferredoxin and CO_2_ are produced in equimolar quantities by PFOR. Cells alleviate some of the need to utilize acetyl-CoA as an electron acceptor by transferring electrons from NADH to ferredoxin, thereby increasing ATP yields [33]. In contrast to gluconate and glucose, the measured H_2_:CO_2_ for galacturonate fermentations was 0.83. This, in conjunction with the very low level of butyrate production during growth on galacturonate, suggests the electron flow through the NADH-ferredoxin oxidoreductase was reversed when compared to growth on glucose and gluconate. The data are consistent with metabolism of galacturonate via the Ashwell pathway as presented in Figure 1. There is equal oxidation and reduction of NADH by altronate oxidoreductase and glyceradehyde-3-P dehydrogenase, leaving reduced ferredoxin generated by PFOR as the only source of reductant for biosynthetic reactions.

**Table 1:** **Calculated ATP and reductant yields, and measured metabolites for growth on different substrates in bioreactors**

	**Import to Acetyl-CoA**				**Acid Production**					
	**ATP**	**NADH**	Fdred*	**AC:BU** ^**‡**^	**ATP**	**NAD** ^**+**^	**NetATP**	**H**_**2**_ **:CO**_**2**_^**¥**^	**H**_**2**_	**NetR.E.** ^**#**^
**Glucose**	*2*	*2*	*2*	**0.82(±0.01)**	*1.45*	*1.10*	*3.45*	**1.15(±0.01)**	*2.30*	*0.60*
**Gluconate**	*0.667*	*1*	*2*	**3.89(±0.05)**	*1.80*	*0.41*	*2.47*	**1.09(±0.01)**	*2.18*	*0.41*
**Galacturonate**	*0.667*	*0*	*2*	**36.79(±5.44)**	*1.97*	*0.05*	*2.64*	**0.83(±0.03)**	*1.66*	*0.29*

Measured values are bold and represent two biological replicates.

Theoretical calculations are italicized.

*Reduced ferredoxin.

^‡^Acetate:Butyrate.

^¥^Measured during maximal gas production.

^#^Net reducing equivalents (NADH + Fdred-NAD^+^-H_2_) available for biosynthetic reactions.

ATP yields were calculated for each of the substrates using the theoretical ATP yield for transport and metabolism to acetyl-CoA combined with the ATP production from conversion of acetyl-CoA to acids using the measured acetate:butyrate ratios. There is an additional ATP burden for import of gluconate and galacturonate via proton symporters when compared to the phosphotransferase system (PTS) used for glucose. Proton symporters use the transmembrane proton gradient, which in *C. acetobutylicum* is maintained by the reversal of the F_0_F_1_ ATP synthase to pump protons out of the cell at stoichiometries of 2–3 H^+^ per ATP [34,35]. It can be calculated from this that the import of one galacturonate or gluconate via a proton symporter costs the cells 0.33 – 0.5 ATP (for this study’s calculations 0.33 ATP was used). Glucose transport, in comparison, relies on the more efficient PTS, which couples glucose transport and phosphorylation. This coupling requires phosphoryl transfer from phosphoenolpyruvate to glucose, yielding pyruvate and glucose-6-P at a cost roughly equivalent to one ATP. In the ED pathway, 2-keto-3-deoxygluconate 6-phosphate aldolase converts KDG to glyceraldyhde-3-P and pyruvate. Only the 3 carbons of glyceraldehyde-3-P pass through lower glycolysis, yielding two ATPs. The two ATPs, coupled with the ATP required for transport and for phosphorylation by 2-keto-3-deoxygluconokinase, create a net ATP production for metabolism of gluconate or galacturonate to acetyl-CoA of approximately 0.667. Glucose is transported by the more efficient PTS and all carbon is processed via lower glycolysis netting two ATPs during uptake and metabolism to acetyl-CoA. The calculations indicate cells grown on gluconate and galacturonate could be starved for ATP when compared to cells grown on glucose; however they produce fewer reducing equivalents, thereby alleviating the burden of using acetyl-CoA as an electron acceptor. In the case of galacturonate nearly all acetyl-CoA was converted to acetate yielding an additional 1.97 ATP per galacturonate. Cells used acetyl-CoA as an electron acceptor during growth on gluconate, producing intermediate levels of butyrate and yielding 1.8 ATP/gluconate during acid production. Glucose fermentation produced the highest concentrations of butyrate and only 1.45 ATP/glucose was calculated to be produced during acid formation. Table 1 shows comparisons of ATP yields calculated using the information above. The comparisons indicate gluconate and galacturonate fermentations were somewhat able to compensate for low ATP yields from upper metabolism by converting more acetyl-CoA to acetate when compared to glucose fermentation.

### Manipulation of metabolic output and growth rate

**Figure 2:**
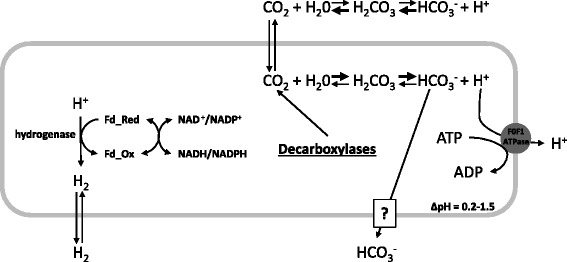
**Possible fate of gases produced during fermentation.** CO_2_ freely diffuses across the cell membrane and reacts with water to form carbonic acid, which dissociates to form bicarbonate and a H^+^. The high pH inside the cell relative to the medium shifts this equilibrium towards bicarbonate, while the opposite occurs extracellulary. Excess H^+^ are pumped out of the cell at the cost of ATP or are reduced to H_2_ by the hydrogenase. Bicarbonate may be transported out of the cell or used in biosynthetic reactions. Adapted from Dixon et al. [38].

Static fermentations can become supersaturated with CO_2_ and H_2_, which may adversely affect metabolism [36-39]. It has been postulated that excessive H_2_ leads to inhibition of the hydrogenase. Also, elevated CO_2_ levels have been shown to inhibit anaerobic yeast and *C. sporogenes* fermentations, presumably by acting as a membrane uncoupler (Figure 2) [38,40]. The uncoupling effect of CO_2_ occurs due to a pH difference between the cytoplasm and extracellular environment, and in *C. acetobutylicum* the ΔpH has been shown to range from approximately 0.2 to 1.5 during acidogenic growth [34,41,42].

Dissolved CO_2_ is in equilibrium with carbonic acid and bicarbonate, with the relative concentrations of each species being highly dependent upon pH. In the low pH of the extracellular environment, the equilibrium shifts towards CO_2_ which can freely cross cellular membranes [38,40]. The relatively high pH inside the cell shifts the equilibrium from CO_2_ towards bicarbonate. As a result, CO_2_ produced intracellularly during metabolism and CO_2_ that diffuses into the cell due to higher extracellular CO_2_ concentrations, can form carbonic acid which dissociates to bicarbonate and a proton, neither of which freely diffuses across the membrane. Intracellular bicarbonate can be used in biosynthetic reactions or is possibly transported out of the cell [40]. The hydrogenase reaction also affects intracellular pH because protons are reduced to H_2_, which can diffuse across the cellular membrane [43]. Reduction of dissolved hydrogen increases hydrogen production from the hydrogenase, presumably due to reducing product inhibition as governed by Le Chatelier's principle [32,44-46]. Reducing dissolved CO_2_ and H_2_ in cultures should therefore lessen the need to expend energy for ΔpH maintenance and improve metabolic efficiency through increased hydrogenase activity (proton removal) and decreased CO_2_ membrane uncoupling.

Beyond potential ΔpH maintenance advantages, differences in available reductant during fermentation of glucose, gluconate, and galacturonate suggested that reducing dissolved hydrogen concentration could impact the cultures differently. Increasing hydrogenase activity through reduction of dissolved H_2_ reduces the need to use acetyl-CoA as an electron acceptor. For substrates such as glucose and gluconate, where the hydrogenase seems to be saturated and there is substantial production of butyrate, increasing hydrogen production should have a positive impact on growth beyond the improved proton removal discussed above. Conversely, for cultures grown on more oxidized substrates such as galacturonate, where biosynthetic reactions compete with the hydrogenase for reductant, increasing hydrogen production could have a negative impact on growth.

Growth was compared in static and agitated cultures in order to begin elucidating the effects of reducing dissolved H_2_ and CO_2_ concentrations. It was presumed that the agitated cultures had relatively low dissolved gas levels, similar to previous experiments [47]. The data shown in Figure 2 indicate that growth rate and maximal culture density were increased by agitation for cells grown on 0.5% glucose or 0.5% gluconate, providing evidence that depletion of dissolved H_2_ and CO_2_ has a positive impact on growth. The growth rate and maximal cell density for cultures grown on 0.5% galacturonate were relatively unaffected by agitation, suggesting any potential benefit of removing CO_2_ was negated due to loss of reducing power provided by H_2_. Residual galacturonate was present in cultures grown on 0.5% galacturonate, indicating the cells were either inhibited by fermentation products or unable to uptake low concentrations of galacturonate. The incomplete fermentation made it difficult to discern if there was a growth advantage in the static or agitated cultures.

**Figure 3:**
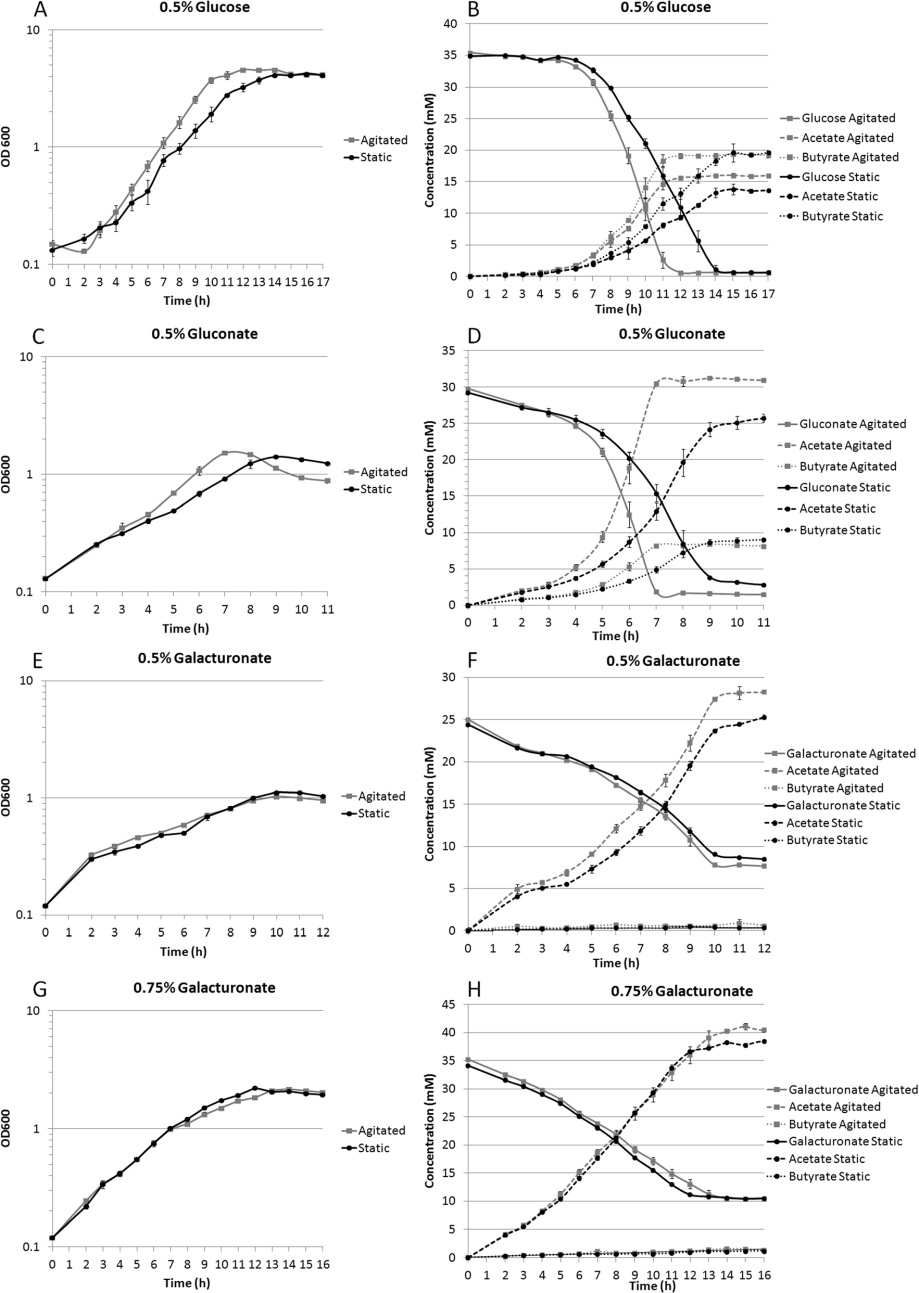
**Growth and metabolic profiles of static verses agitated cultures.** Growth curves **(A, C, E, G)** and HPLC analysis **(B, D, F, H)** of cultures containing 0.5% glucose **(A, B)**, 0.5% gluconate **(C, D)**, 0.5% galacturonate **(E, F)**, and 0.75% galacturonate **(G, H)**. Growth curve graphs for cultures grown with agitation are shown with grey squares and solid grey lines, and static cultures with black circles and solid black lines. In HPLC graphs hexose and hexose derivatives are shown with solid lines, acetate with dashed lines, and butyrate with dotted lines. Error bars represent +/− one standard deviation from three replicate cultures.

To address incomplete galacturonate utilization, cultures grown on 0.75% galacturonate were examined. While more residual galacturonate remained after the fermentation was complete, a larger amount of galacturonate was utilized and a higher acetate concentration was reached compared to cultures grown on 0.5% galacturonate, suggesting product inhibition was not the primary cause of incomplete fermentations. Furthermore, static fermentations of 0.75% galacturonate appeared to have a slight growth advantage over agitated cultures above an OD_600_ of approximately one; this indicates the agitated cells may be starved for reductant at higher cell densities. Agitation increased production of oxidized products for all substrates tested, as indicated by the higher titers of acetate shown in Table 2. There were little to no differences in final butyrate concentrations which resulted in increased acetate:butyrate ratios (Table 2) for agitated cultures. If improved ATP economics was the main contributing factor to increased growth rates for agitated cultures, then there would have been an increase in maximal cell density coupled with a decrease in product yield. Higher product yields (Table 2) and maximal cell densities (Figure 3) for agitated vs. static cultures grown on glucose and gluconate indicate agitation increased metabolic efficiency beyond simply improving ATP yields through increased conversion of acetyl-CoA to acetate or reducing ATP requirements for ΔpH maintenance. These results are consistent with observations in other organisms, where elevated CO_2_ concentrations inhibit and/or promote decarboxylase and carboxylase enzymes respectively, which in turn could lead to changes in metabolic efficiency [48]. Since C1 units in *C. acetobutylicum* are derived primarily from the carboxyl group of pyruvate and not CO_2,_ there may be limited inhibition of metabolism by removal of CO_2_ from cultures [49]. One CO_2_ requiring enzyme that is integral to *C. acetobutylicum’s* central metabolism is pyruvate carboxylase, and it has been proposed that its inhibition could direct flux away from metabolic precursors in the TCA and increase flux to acetyl-CoA derived products [49]. Enhancement of PFOR by CO_2_ removal could improve metabolic efficiency and direct more carbon to acetyl-CoA. Additionally, enhancement of decarboxylating enzymes (isocitrate dehydrogenase and α-ketoglutarate dehydrogenase) of the oxidative branch of the TCA by CO_2_ removal could also provide a growth advantage and help compensate for potential oxidative TCA inefficiencies from pyruvate carboxylase inhibition.

**Table 2:** **End point HPLC analysis of substrates and products from fermentations shown in Figure** 3

	**Substrate used (mM)**	**Acetate (mM)**	**Butyrate (mM)**	**Ac:Bu**	**ATP/Substrate**	**Portion of C in products**
Glucose Static	**34.4(±0.1)**	**13.4(±0.4)**	**19.1(±0.3)**	0.70	*3.41*	0.751
Glucose Agitated	**34.9(±0.1)**	**15.8(±0.3)**	**19(±0.3)**	0.83	*3.45*	0.771
Gluconate Static	**28.2(±0.3)**	**29.2(±1.8)**	**9(±0.7)**	3.24	*2.43*	0.840
Gluconate Agitated	**28.5(±0.6)**	**31.2(±2.1)**	**8.9(±0.7)**	3.50	*2.44*	0.860
0.5% Galacturonate Static	**15.9(±0.2)**	**25.3(±0.3)**	**0.4(±0.0)**	70.3	*2.65*	0.817
0.5% Galacturonte Agitated	**17.3(±0.1)**	**28.3(±0.0)**	**0.6(±0.1)**	47.1	*2.65*	0.850
0.75% Galactuornate Static	**24 (±0.1)**	**39.3(±0.2)**	**1.4(±0.0)**	27.7	*2.63*	0.879
0.75% Galacturonate Agitated	**24.8(±0.2)**	**41.3(±0.7)**	**1.5(±0.0)**	27.5	*2.63*	0.895

Measured values are bold and represent three biological replicates.

Theoretical calculations are italicized.

### Towards homoacetic fermentation

**Figure 4:**
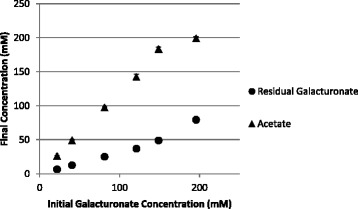
**Correlations between initial galacturonate concentrations and residual galacturonate and acetate concentrations.** Results are from three replicate cultures and error bars represent +/− one standard deviation.

The nearly homoacetic fermentation of galacturonate by *C. acetobutylicum* indicated that fermentation of oxidize feedstocks could be a viable route for biologically produced acetate. To determine the level of acetate the cells could produce, acetate formation was measured for static cultures grown on increasing concentrations of galacturonate without any other modifications to the media or growth conditions. Final acetate concentrations shown in Figure 4 increased with initial galacturonate concentration with the fermentations of 5% galacturonate producing approximately 200 mM acetate, which is equivalent to 1.2% W/V. Residual galacturonate also increased with initial galacturonate concentration indicating that the cause of incomplete fermentations is probably multifaceted. The concentrations of acetate reached in these fermentations were not sufficient to be an industrial source of acetate but there was no attempt to optimize fermentations beyond increasing substrate concentration. Optimization of *C. acetobutylicum* fermentations for acetate production has not been well studied. Further efforts are needed to manipulate growth conditions and/or apply genetic engineering strategies to reach sufficient acetate titers to be competitive with other processes.

## Conclusions

Recent advances in “omics” technology, systems biology, synthetic biology, and metabolic engineering have allowed for the development of tailored microbes for the synthesis of biofuels by assembling natural and *de novo* pathways that redirect available carbon to desired products [50-53]. Far less attention has been paid to engineering organisms that completely utilize all available carbon sources. Comprehensive knowledge of carbohydrate catabolism, as well as the influence of various carbohydrates have on overall metabolism, is required to fully develop such a microbial factory. To this end, we characterized the catabolic pathways and metabolic impact of the hexose derivatives gluconate and galacturonate in *C. acetobutylicum*.

This work presents a complete metabolic network for metabolism of glucose, gluconate, and galacturonate in *C. acetobutylicum*. The network should facilitate similar reconstructions in other organisms and provides a further understanding of the pathways involved in metabolism of carbohydrate mixtures, such as slop food waste. Comparisons of metabolic output between fermentations of glucose, gluconate, or galacturonate indicated cells grown on glucose and gluconate had an excess of reducing power but cells grown on galacturonate seemed to be limited by available reductant. Further evidence was demonstrated by comparisons of growth rates on agitated and static cultures. Glucose and gluconate grown cells benefited from agitation, presumably as a result of improved ATP economics from removing dissolved H_2_ and/or CO_2_. In contrast, cells grown on galacturonate had a growth advantage when the cultures were static, which could be due to increasing availability of reductant via product inhibition of the hydrogenase. The growth advantage from agitating cultures grown on glucose or gluconate could not fully be explained by improved efficiencies of ATP production (increased acetate:butyrate ratio) or reduction of ATP required for ΔpH maintenance. It is possible that elevated H_2_ or CO_2_ concentrations inhibit other aspects of metabolism, as is the case for CO_2_ inhibition of decarboxylase reactions [48]. From the data presented it is clear that metabolic effects due to dissolved H_2_ and CO_2_ are complex and will require further study to fully elucidate the role each gas has on metabolic efficiency and output.

The pathway used to metabolize a substrate, as well as its oxidation state, have long been known to have a profound impact on the products of microbial fermentations [10]. ABE fermentations with reduced feedstocks such as mannitol and glucose/glycerol mixtures have been shown to produce more butanol than fermentations of glucose [33]. Addition of glycerol has a confounded effect on product output. Hydrogenase activity decreases despite the additional requirement to dispose of electrons and the cells compensate for this by increasing butanol production [33]. The reduction of hydrogenase activity results in electron flow from reduced ferredoxin to NAD^+^, mediated by ferredoxin-NAD reductase, which was shown to be a distinct enzyme from the NAD-ferredoxin reductase [43]. In the current study NAD-ferredoxin reductase and ferredoxin-NAD reductase activities were not measured, but it is probable that different enzymes mediated NAD/ferredoxin electron transfer during growth on galacturonate when compared to glucose or gluconate grown cells, similar to fermentations containing glycerol.

Reduced products are often the most relevant substrates for the biofuel industry, therefore an in depth understanding of an oxidized feedstock’s metabolism could reveal opportunities to engineer organisms with the capacity to produce larger amounts of desirable products. Research on *C. acetobutylicum* has historically been centered on strategies for increasing butanol yield through genetic engineering and manipulation of fermentation conditions [54]. More recent publications have emerged in the literature describing the production of other commercially viable products, such as isopropanol and 2,3 butanediol from *C. acetobutylicum* [55,56]. To enhance biofuel production, strategies to reduce acetone production by distruption of acetoacetate decarboxylase (*adc*) and acetoacetyl-CoA:acyl-CoA transferase (*ctfAB*) have been attempted [57,58], as well as conversion of acetone to a potential biofuel [59-61]. The knowledge provided in the current study could be used to target gene deletions for disruption pathways required for metabolism of oxidized feedstocks. It is probable that these deletions would reduce formation of undesirable products, thereby improving conversion of inexpensive feedstocks to biofuels.

As genetic engineering strategies for *C. acetobutylicum* improve, it can be expected that an expanded list of products will be able to be produced by the organism. This, coupled with the ability of the organism to use a wide range of substrates, makes it an attractive candidate for conversion of heterogeneous biomass to useful products. The ability to direct metabolic flow through desired pathways is essential for production of commodity chemicals. The work presented in this study provides additional knowledge about the metabolic pathways used for fermentation of oxidized feedstocks and their impact on metabolic output. Production of desired products in the future could be improved by utilizing this information to direct carbon or electron flow in *C. acetobutylicum.*

## Methods

### Bacterial strain propagation

*C. acetobutylicum* ATCC 824 was propagated on clostridial growth medium (CGM) as previously described [3]. Each liter of CGM contained: KH_2_PO_4_, 0.75 g; K_2_HPO_4_, 0.75 g; MgSO_4_•H_2_O, 0.4 g; MnSO_4_•H_2_O, 0.01 g; FeSO_4_•7H_2_O, 0.01 g; NaCl, 1.0 g; asparagine, 2.0 g; yeast extract, 5.0 g; (NH_4_)_2_SO_4_, 2.0 g; substrate, 5 g (Note: 5 g substrate refers to 0.5% media; for different percentages the amount of substrate was changed accordingly). For NMR analysis the medium contained 0.5% D-[1-^13^C] gluconate (Omicron Biochemicals, Inc.).

### Growth in bioreactor

750 mL cultures of CGM, supplemented with 0.5% glucose, gluconate, or galacturonate were prepared in a 4-vessel DasGip bioreactor system (Eppendorf). The temperature was maintained at 37°C with agitation via a Rushton impeller at 400 RPM. OD_600_ for each vessel was recorded every 30s with a DasGip probe (10 mm path length). Redox potential and pH for each vessel were similarly recorded using Broadly-James redox probes and Mettler-Toledo pH probes, respectively. Each bioreactor was inoculated with 10 mL injections of cultures grown overnight on CGM containing 0.5% glucose.

### Metabolite analysis

Duplicate cultures of *C. acetobutylicum* were grown in a four-vessel bioreactor as described above on CGM, supplemented with 0.5% of glucose, gluconate, or galacturonate. Next, 1.0 ml of culture was removed two hours after inoculation and then at 1-h intervals. The sample was clarified by centrifugation at 13,000 × g in an Eppendorf 5415D microcentrifuge for 2 min. The supernatant was then filtered through a 0.2 μm syringe filter and stored at −80°C. HPLC analysis of metabolites was performed on an Agilent 1200 equipped with a refractive index detector and an Aminex HPX-87H cation exchange column (300 × 7.8 mm i.d. × 9 μm) (Bio-Rad) as previously described [62]. Samples (100 μl) were injected into the HPLC system and were eluted isocratically with a mobile phase of 3.25 mM H_2_SO_4_ at 0.6 ml/min and 30°C. Quantification was based on an external calibration curve using pure known components as standards.

### Headspace analysis

DasGip bioreactors were setup with an inflow of 0.1 L/min N_2_ gas. Bioreactor gas outflow was collected through a gas flow manifold (Valco Instruments EMT EMTCA-CE) connected to a micro-GC (Agilent 3000A MicroGC). Each reactor’s GC profile was measured every 15 min. from prior to inoculation through conclusion of exponential growth phase.

### Growth curve determination for static vs. agitated cultures

Three replicate flasks of both static and agitated CGM (100 mL, supplemented with one of the following: 0.5% glucose, 0.5% gluconate, 0.5% galacturonate, or 0.75% galacturonate) were prepared in an anaerobic chamber (Coy Labs). Magnetic stir bars and magnetic stir plates (IKA Color Squid S1) were used to agitate cultures as indicated. Each flask was inoculated with cultures grown overnight on CGM so the OD_600_ as measured by an Ultrospec 10 (Amersham Biosciences) was approximately 0.12. The OD_600_ for all cultures was measured and record hourly thereafter until cultures reached stationary phase.

### NMR analysis

Samples for NMR analysis were prepared in 85% water with 15% D_2_O. All NMR spectra were recorded at 25°C on a Bruker AVANCE_III spectrometer. ^1^H NMR spectra were recorded at 600.13 MHz with the solvent suppression technique. ^13^C-NMR spectra were recorded at 125.75 MHz with H-1 inversed gated decoupling (without NOE). Between 500–2048 scans were collected to obtain the desired resolution.

## Abbreviations

ED: Entner-doudoroff
PFOR: Pyruvate-ferredoxin oxidoreductase
KDG: 2-keto-3-deoxy-gluconate
KDPG: 2-keto-3-deoxy-6-phospho-D-gluconate
PTS: Phospho transferase system
TCA: tricarboxylic acid cycle
